# Evaluation of the biocontrol efficacy of a *Serratia marcescens* strain indigenous to tea rhizosphere for the management of root rot disease in tea

**DOI:** 10.1371/journal.pone.0191761

**Published:** 2018-02-21

**Authors:** Gargee Dhar Purkayastha, Preeti Mangar, Aniruddha Saha, Dipanwita Saha

**Affiliations:** 1 Department of Biotechnology, University of North Bengal, Siliguri, West Bengal, India; 2 Department of Botany, University of North Bengal, Siliguri, West Bengal, India; Universita degli Studi di Pisa, ITALY

## Abstract

The aim of the present study is to evaluate plant growth promoting and biocontrol efficacy of a *Serratia marcescens* strain ETR17 isolated from tea rhizosphere for the effective management of root rot disease in tea. Isolated bacterial culture ETR17 showed significant level of *in vitro* antagonism against nine different foliar and root pathogens of tea. The phenotypic and molecular characterization of ETR17 revealed the identity of the bacterium as *Serratia marcescens*. The bacterium was found to produce several hydrolytic enzymes like chitinase, protease, lipase, cellulase and plant growth promoting metabolites like IAA and siderophore. Scanning electron microscopic studies on the interaction zone between pathogen and antagonistic bacterial isolate revealed severe deformities in the fungal mycelia. Spectral analyses (LC-ESI-MS, UV-VIS spectrophotometry and HPLC) and TLC indicated the presence of the antibiotics pyrrolnitrin and prodigiosin in the extracellular bacterial culture extracts. Biofilm formation by ETR17 on polystyrene surface was also observed. *In vivo* application of talc-based formulations prepared with the isolate ETR17 in tea plantlets under green house conditions revealed effective reduction of root-rot disease as well as plant growth promotion to a considerable extent. Viability studies with the ETR17 talc formulation showed the survivability of the isolate up to six months at room temperature. The sustenance of ETR17 (concentration of 8-9x10^8^ cfu g^-1^) in the soil after the application of talc formulation was recorded by ELISA. Safety studies revealed that ETR17 did not produce hemolysin as observed in pathogenic *Serratia* strains. The biocontrol strain reported in this study can be used for field application in order to minimize the use of chemical fungicides for disease control in tea gardens.

## Introduction

North-East India especially the sub-Himalayan region of West Bengal and adjoining state of Assam is the largest tea growing region of India. High humidity is required for the growth of tea leaves. High humidity makes the tea plants prone to attack by several fungi as fungi thrive well in humid atmosphere. The varying conditions of climate, soil and several environmental stresses required for tea plantation makes it vulnerable to attack by several fungal pathogens [1–3]. Over the years, plant disease management strategies were mostly based on the use of chemical fungicides. But the uncontrolled and persistent use of chemicals results in harmful residual effect on plants, natural soil microflora and human health. Pathogenic fungus may also develop resistance against fungicides [4, 5, 6]. The rhizosphere inhabiting antagonistic bacteria serve as a promising alternative to synthetic fungicides for controlling several plant diseases without disturbing the natural ecosystem [7, 8, 9].

A comprehensive research in the past few decades has highlighted the role of both gram-positive and gram-negative bacterial strains as promising biological control agents [10–13]. Among the antifungal metabolites produced by biocontrol agents to combat phytopathogens, antibiotics are the most widely studied compounds [14]. In several instances, antibiotics such as pyrrolnitrin, 2, 4- diacetylphloroglucinol (DAPG), prodigiosin, pyoluteorin, phenazine-1-carboxylic acid (PCA), paenimyxin and several others have been shown to be particularly effective in suppressing plant pathogens and reduces the disease incidences they cause [15–20]. The expression and secretion of lytic enzymes like chitinase, cellulase, protease and DNase produced by soil bacteria also result in direct suppression of pathogenic activities by hydrolyzing several polymeric compounds [10, 21, 22]. Furthermore, bacterial siderophores play a major role in plant disease suppression by iron sequestration since fungal siderophores have lower affinity towards iron available in soil [23, 24]. Literature reports indicate that biological control of plant pathogens is a complex process and antagonism against target fungus is often multifaceted [5].

Root associated bacteria can influence plant growth and development and overall plant health [25–27]. Additionally, extensive research has been carried out to provide effective means of *in vivo* application of biocontrol agents in the form of stable bioformulations and their sustenance in soil environment for enhanced disease control and plant growth promotion [2, 28, 29]. Despite the apparent successes associated with the use of biocontrol agents for combating the plant diseases, their chemical counterparts still succeed over them. The reason lies with the fact that, under field conditions a biocontrol agent largely depends upon its interactions with the natural microbiota, ability to compete for nutrients, effective root colonization and adapting to the changes in environmental conditions [30–32].

The present study aims at the use of a potential antagonistic bacterial strain indigenous to tea rhizosphere for controlling a severe root disease of tea. In an attempt to fulfill the objectives of this study, a potential strain of *S*. *marcescens* with multiple bicontrol attributes was isolated from tea rhizosphere and characterized. Biocontrol experiments were conducted against *Rhizoctonia* root rot in experimental tea seedlings.

## Materials and methods

### Pathogens

Nine different fungal strains causing several foliar and root diseases in tea (*Camellia sinensis*) were used in the present study. The pathogens *Curvularia eragrostidis* (ITCC 4150.2k), *Pestalotiopsis theae* (PT01), *Colletotrichum camelliae* (CC01), *Lasiodiplodia theobromae* (ITCC 5446.02) and *Rhizoctonia solani* (ITCC 5995.05) were isolated from affected tea plants during earlier studies [1, 33–36] and the identities of the ITCC strains were authenticated by Indian Agricultural Research Institute, New Delhi. *Sphaerostilbe repens* (SR-01), *Fomes lamaoensis* (FL-01), *Poria hypobrunae* (PH-01) and *Ustulina zonata* (UZ-01) were procured from Tea Research Association, Tocklai Experimental Station, Jorhat, Assam.

### Plant material

One month old disease free tea (*Camelia sinensis*) seedlings of TS-520 variety were obtained from Gayaganga tea estate located in Darjeeling district of West Bengal during April. The soil of the tea estate was sandy loam(pH of 4.8).The organic carbon content was moderate at 20.9 g/kg of soil with electrical conductivity of 0.03 dsm^-1^. The available N-P-K content of the soil was 122.45 kg/ha-^1^, 22.12 kg/ha^-1^and 261.33 kg/ ha^-1^ respectively [37]. The temperature prevailing was 27 ± 2°C with average rainfall of 94 mm. The procured seedlings were maintained in earthen pots of 16cm diameter and 11cm height in the experimental garden of the Department of Botany, University of North Bengal under normal light and temperature. The tea plantlets were used for experimental purpose after 30 days of acclimatization.

### Isolation of bacteria

Rhizosphere soil samples were collected from fifteen different tea gardens located in West Bengal and Assam. The tea plantations of the sampling sites selected for the study remained free from any prior application of biocontrol agents. Roots along with adherent soil were collected from healthy plants aseptically in sterilized bags and transported to the laboratory within an hour. Isolation of bacteria was done following previously described methods [38, 39]. Two hundred bacterial strains were isolated in nutrient agar medium (HiMedia Laboratories Pvt. Ltd., Mumbai) and maintained in nutrient agar (NA) slants. After incubation for 72 h at 30°C in inverted position, isolated single colonies were picked from spread plates and pure cultures were obtained on NA slants. The strains were subsequently maintained in NA slants with periodic transfers to fresh medium.

### Test for *in vitro* antagonism

All isolated strains were initially screened *in vitro* by dual culture technique for the presence of antagonistic activity against *L*. *theobromae* on potato dextrose agar (PDA) medium [39]. A 4 mm diameter mycelial disc of the fungal pathogen was collected from advancing zone of hyphae growing in PDA plates and inoculated at the centre of a 9 cm diameter petriplate containing PDA medium. The bacterial isolate was streaked at a distance of 2–3 cm from the centre in semi-circular/circular pattern. The plates were incubated at 30°C and checked daily for inhibition until the fungal growth on the control plate (inoculated only with the pathogen) reached the edge of the plate. Inhibition of fungal growth along the bacterial line of streaking indicated antagonistic activity of the isolated bacterial strain. Based on the initial screening (S1 Table), the most promising isolate ETR17 was selected for further study. The isolate was further tested for antagonism against eight other fungal pathogens following the same method. Percentage (%) of inhibition of the mycelial growth of test pathogens was calculated as [(Diameter of pathogen growth in control plate (mm)-Diameter of pathogen growth in presence of antagonist(mm)/Diameter of pathogen growth in control plate (mm)] X 100 [38, 39]. The dual culture tests were performed in three replications and the data was averaged. *L*. *theobromae* was chosen for initial screening because it is the most common pathogen of tea and can attack all parts of plant [40–42].

### Characterization of ETR17

Phenotypic traits of the bacterial isolate ETR17 were examined based on the biochemical, physiological and morphological characteristics [43, 44]. The culture characteristics such as shape, size, colony nature and pigmentation of 3 day old bacterial culture were examined. The isolate was further tested for gram staining, indole, methyl red, Voges Proskauer, citrate utilization, catalase, oxidase, gelatin and starch hydrolysis, oxidation-fermentation, nitrate reduction, decarboxylation of ornithine, arginine and lysine, utilization of different carbon sources such as glucose, lactose, mannitol, sucrose, fructose, arabinose, adonitol, cellobiose, inositol, rhamnose, raffinose, sorbitol, trehalose and xylose and growth at four different temperatures. Results of these tests were scored as either positive or negative.

Further characterization of the antagonistic isolate was performed using 16SrRNA gene sequence. In brief, the total genomic DNA was isolated from ETR17 by CTAB method [45]. Amplification of the 16S rRNA gene was performed in 25μl reaction using Universal primers following specific conditions on a thermal cycler (Applied Biosystems GeneAmp PCR 2400) [39]. The amplified product was purified using PCR purification Kit (Bangalore Genei, India) and sequenced at Bangalore Genei Sequencing Services. The 16S rRNA gene sequence was thereafter deposited in the NCBI GenBank and accession number (JX566992) was assigned. A similarity search of the sequence was carried out and compared with the 16S rRNA sequences of related species available in GenBank databases using the BLAST search program of the National Center for Biotechnology Information (NCBI) [46]. The 16S rRNA gene sequences of the bacterial strains having similarity ranging from 98% to 100% with the target sequence were used for sequence alignment.

### Studies on the interaction between biocontrol isolate ETR 17 and pathogen

The fungal mycelia of the pathogen *R*. *solani* collected from the interaction zone of the bacterium ETR17 and the pathogen in dual culture PDA plates were used as samples for microscopic studies. The sample was prepared following the method of Saha et al [39] and viewed under a Scanning electron microscope [Model: Hitachi S-530 (Japan) 1986].

### Detection of extracellular hydrolytic enzymes produced by ETR17 isolate

Chitinase activity was detected by following the method of Bargabus et al [47]. Positive result was indicated by the presence of non fluorescent lytic zones around the wells under UV light. Production of cellulase and pectinase enzymes was detected following the method of Cattelan et al [48] with some modifications. For assessing cellulase activity, bacterial culture supernatant was added to 4mm agar cups of M9 agar medium [49] supplemented with 10 g L^-1^ cellulose and 1.2 g L^-1^ yeast extract. The plate was incubated at 30°C for 5–6 days and thereafter stained with 0.1% Congo red solution overnight and destained thrice with 1M NaCl at 2hrs interval. Formation of clear halo indicated positive result. Pectinase activity was tested in the same medium by amending with 10 g L^-1^ pectin instead of cellulose. The plate was streak inoculated and incubated for 2 days at 30°C, then it was flooded with 2M HCl. A positive result was indicated by visible clear halos around the colonies. Lipase and protease activities were tested following the method of Smibert and Kreig [50].

### Testing for plant growth promoting factors and other biocontrol traits

Production of the phytohormone IAA was investigated spectrophotometrically by using the method of Patten and Glick [51]. The absorbance maxima of IAA in the bacterial culture supernatant were recorded at 535 nm and its concentration was determined by comparison with the standard curve generated from different concentrations of IAA (Merck, India). The ability of *S*. *marcescens* strain ETR17 to solubilize phosphate was determined in Pikovskaya’s agar media [52]. Positive result is indicated by the development of a clear zone around the bacterial colonies. Siderophore production was determined using the Universal Chrom azurol S (CAS) assay [53]. Identification of catecholate type of siderophore was carried out following the method of Arnow [54]. Hydroxamate nature was examined by tetrazolium salt test indicated by instant appearance of a deep red colour by addition of siderophore sample to tetrazolium salt under alkaline conditions [55]. Test for HCN production was carried out by the method of Bakkers and Schippers [56] where positive result was indicated by the color shift of a filter paper strip immersed in picric acid from yellow to red. Biofilm formation on microtiter plates by the biocontrol bacterial isolate was assessed in two different medium [Luria Bertani (LB) broth and M9 Yeast extract (M9YE) broth [57].

### Extraction, detection and purification of antifungal antibiotic from antagonistic bacterium *S*. *marcescens* strain ETR17

Bacterial strain ETR17 was inoculated into semi-solid pigment producing media [58] by pour plate method and incubated at 30°C for 8 d. The total content (180 mL) was crushed in a blender and extracted with 250 mL of 80% aqueous acetone for 24 hours in an orbital shaker [59]. Agar was removed by centrifugation at 15,000 rpm for 20 min at 10°C and the supernatant containing antibiotics was condensed at 40°C in a rotary vacuum evaporator (Eyela CCA-1110, Japan). The aqueous concentrate was filtered through cellulose acetate filter paper (Sartorius, pore size 0.2 μm) and 20 mL portions of the filtrate were extracted twice with 2.5 volumes of diethyl ether. The organic phase containing antibiotics were evaporated to dryness *in vacuo* at 30°C and the residue was re-extracted with 30 mL of acetone, and finally condensed under vacuum to obtain a red pasty mass [38].

The crude extract (2.5 g) was dissolved in 10 mL methanol, mixed and dried over silica gel (mesh 60–120) and was subjected to silica gel column chromatography. Elution with 100% petroleum ether and petroleum ether -ethyl acetate (95% to 50%) yielded a total of eleven fractions (F1 to F11) of 100 mL each. Each fraction was vacuum concentrated and tested for antifungal activity. The active fractions F3, F4, F5, F10 and F11 were monitored by thin layer chromatography (TLC). Samples were loaded on TLC sheets pre-coated with Silica Gel 60 F254 and co-chromatographed with standard antibiotics pyrrolnitrin, prodigiosin and phenazine (Sigma-Aldrich). The sheets were developed in benzene:acetic acid (9:1) and viewed under UV (254 nm) light. The antibiotics present in the bioactive fractions were further purified by preparative TLC. For this, large volumes (1 mL) of the bioactive fractions were spotted as before on glass-backed preparative TLC plates (prepared manually by coating with silica gel G. The sheets were developed similarly in benzene: acetic acid (9:1) and the zone corresponding to the R_f_ value obtained on analytical plates were scrapped from the TLC plate, suspended in methanol and centrifuged. The supernatant was dried *in vacuo* and dissolved in methanol. Partially purified compound in methanol was scanned between 200 to 700 nm on a dual beam Varian Cary 50 Bio UV-Visible spectrophotometer (Varian, Australia) along with standard prodigiosin and pyrrolnitrin. Thus two antibiotics were detected by analytical TLC. High performance liquid chromatographic analysis of the antifungal metabolites purified by TLC was performed in Shimadzu SPD-20A, Japan. Fractions which appeared to contain same antibiotics were combined, dissolved in methanol (50μg per mL) and 20 μl was injected into C18 Reverse Phase column (250 x 4.6mm size and 4μm particle size) (Phenomenex, USA). The pump used was LC-20AD (Shimadzu, Japan). The eluent flow rate was adjusted to 1 mL min^-1^ and analyzed isocratically in 100% methanol. Standard antibiotics were used at a concentration of 10 μg mL^-1^. Pyrrolnitrin was detected at 225 nm using a D2 detector (Prominence, Shimadzu, Japan) and prodigiosin was detected at 536 nm using tungsten (W) detector (Prominence, Shimadzu, Japan).

Presence of pyrrolnitrin was further confirmed by LC-ESI-MS analysis using a gradient elution program with solvent A (methanol) and solvent D (ammonium acetate buffer, pH 6.5): 50% solvent A and 50% solvent D from 0 to 10 min; 70% solvent A, 30% solvent D at 10 min and 80% solvent A, 20% solvent D till 30 min at a flow rate of 0.8 mL min^-1^ and 254 nm. A 20 μl sample was injected into the column without any dilution. The column used was Thermo ODS-2 (250 x 4.6 mm size and 5 μm particle size) (Thermo, India). The electrospray ionization mass spectra were recorded on a Thermo LCQ Advantage Max (Thermo, India) with the following specifications: Source voltage 5.3V, source current 80.0 μA, capillary voltage 3.0 V, tube lens offset 5.0 V and capillary temperature of 300°C.

### Test for *in vitro* antifungal activity

Antifungal activity of the crude extract and column eluted fractions of ETR17 was assessed *in vitro* against the test pathogens by agar cup bioassay. For this, PDA medium was autoclaved at 121°C for 15 min, cooled to 45°C and 1 mL of pure sporulated mycelial suspensions ofpathogens were mixed with 19 mL of molten medium, and poured into sterile petriplates of 9 cm diameter. Spore suspension of each pathogen except *R*. *solani* was prepared in sterile distilled water (1 x 10^6^ conidia mL^-1^) from 10–12 day- old PDA cultures of each of the pathogens following the method of Saha et al [1]. In case of *R*. *solani*, mycelium from a 7 day old culture was scrapped lightly in 2 mL of sterile distilled water with inoculation needle and mixed with 18 mL molten medium. The mixture was then poured into sterile petriplates (9 cm diameter) and allowed to solidify.After solidification of the medium in petriplates, wells (4 mm in diameter) were prepared with sterile cork-borer. Crude metabolite or individual column fractions were diluted separately by dissolving 100 μl of it in 1mL methanol and then loaded into the wells. A well in the same plate was loaded with 100% methanol which served as the control. The plates were incubated at 28°C for 2–4 days and diameter of inhibition zones formed around the wells, if any, was measured.

### *In vivo* biocontrol study for reduction of root rot disease in tea using bacterial formulation

#### Preparation of talc-based bacterial formulation

Talc-based formulation was prepared with bacterial strain ETR17 following the method of Nandakumar et al [60]. At first, 48 h old nutrient broth (NB) (HiMedia Laboratories Pvt. Ltd. Mumbai, India) culture of the bacterium was used to inoculate 400 mLof sterile NB and grown at 30°C on a rotary shaker operating at 150 rpm. The culture at its stationary phase of growth was centrifuged at 6000 rpm for 10 minutes and bacterial cells were resuspended in 10 mM phosphate buffer (pH 7.0). The concentration was adjusted to 9x10^8^ colony forming unit (cfu) mL^-1^. Talc powder (used as carrier) was mixed thoroughly with calcium carbonate at the rate of 15 g kg^-1^ for adjusting its pH to 7.0. Next, carboxymethyl cellulose was added to the talc (at 10 g kg^-1^), mixed well and the resultant mixture was autoclaved (for 30 min at 15 psi pressure) twice in two consecutive days. Bacterial culture (400 mL) was added to 1 kg of the carrier-cellulose mixture and mixed well under sterile conditions. The mixture was dried aseptically and packed in sterile polypropylene bags, sealed and stored at room temperature (25± 2°C) for future use.

#### Preparation of fungal inoculum

Pathogen inoculum was prepared following the method of Soares et al [61]. For this, 300 g of rice grains were soaked in 500 mLof distilled water at room temperature for one hour for proper hydration. The excess water was drained off and the grains were distributed equally in three 250 mLconical flasks and autoclaved (for 30 minutes at 15 psi pressure) twice on two consecutive days. Mycelial disc (6 mm) of the pathogen *R*. *solani* excised from actively growing regions of a 7 day old fungal culture in PDA plate was used to inoculate the sterilized rice and incubated for 6–9 days at 28°C until the pathogen mycelia covered all the rice grains. The resulting inoculum of mycelium infested rice grains were added at the rate of 10 g per kg soil in the experimental pots.

#### Biocontrol using talc-based formulation

For *in vivo* biocontrol study, two month old tea seedlings were transferred to pots (10 cm x 15 cm) each containing 2 kg of either sterile or non-sterile garden soil amended with 25% super phosphate: rock phosphate (1:1) fertilizer. Ten grams of talc based formulation was mixed with 100 mL of sterile distilled water and poured into the experimental pots. Altogether 25 plants per treatment were used and 5 plants were uprooted each day for assessment of disease. A set of control pots received no treatment. A fungicide control was included where the pots were treated with 0.1% thiophanate methyl (Biostadt India Ltd., Mumbai). After three days of treatment, inoculation was done with the root rot pathogen *R*. *solani*. For this, the soil was removed carefully, mixed with the rice inoculum and replaced back to the original pots. The whole experiment was done under both sterile and non-sterile soil conditions in order to confirm the biocontrol efficacy of ETR17 and to check the effect of any indigenous microorganism present in the soil in disease control. The soil used had sandy-loam texture and acidic pH of 4.5–5.0. The plants were covered with plastic bags to maintain humid condition and kept in experimental net house under normal light and temperature conditions (light: approximately 13 hours each day; temperature: 22.8 to 32.1°C). The plants were arranged in a randomized block design with five replicates for each assessment and each treatment. The entire experiment was conducted thrice and the results of individual experiments were taken together to determine the mean and expressed as mean disease index (MDI). Assessment of root disease was done at 15, 20, 25, 30 and finally 45 days after pathogen inoculation (dapi). Severity of the symptoms was graded into five disease classes (0–4) [62]. The total of 5 seedlings for each treatment were uprooted carefully and graded as follows: 0 = no disease; 1 = superficial rot affected roots; 2 = moderate rot on roots; 3 = severe rot and 4 = completely damaged roots. Based on the classes, disease index was calculated using the following formula: Disease index = [Ʃ (P x DC) x 100]/ (T x4) where P = plants per class, DC = disease class and T = total number of plants. Percent efficacy of disease control (PEDC) was calculated using the formula: PEDC = [(MDI in untreated control–MDI in treated plants)/MDI in untreated control] x100 [38].

#### Determination of the survivability of ETR17 in talc formulation

The population of *S*. *marcescens* strain ETR17 was assessed in the talc formulated product at an interval of one month for one year. One gram samples of talc formulation were collected aseptically and suitable dilutions (10^−1^ to 10^−5^) were prepared in sterile distilled water. An aliquot of 100 μl of the individual dilutions were inoculated in NA medium by spread plate technique. The visible number of colonies formed after incubation at 30°C for 24 h were recorded and the bacterial population was expressed as cfu g^-1^. The plate count was recorded as mean of three replicates and plotted against time as log cfu g^-1^.

#### Sustainability assay of ETR17 in the rhizosphere by ELISA

The sustainability of ETR17 in the rhizosphere when applied as talc based formulation was determined by indirect ELISA [63, 34]. For preparation of bacterial antigen, ETR17 isolate was grown in NB for 48 hours at 30°C on a rotary shaker. The resulting cell culture was centrifuged at 10,000 rpm for 10 min; the pellet was suspended in sterilized 0.15M phosphate buffer saline (PBS) (pH 7.2) and washed thrice by centrifugation using previous conditions. The pellet was resuspended in PBS and bacterial population was inactivated by addition of 1% formaldehyde. The concentration of bacterial suspension was adjusted to an OD_545 nm_ of approximately 1.0 [64]. For raising antiserum against bacterial antigen, equal volume of the cell suspension and Freund’s incomplete adjuvant (Bangalore Genei, India) were mixed and used as antigens for immunizing New Zealand white male rabbit. Antisera were collected using standard methods at regular intervals [34] and stored in sterile cryo-vials at -20°C until required.

For soil antigen preparation, rhizosphere soil samples were collected from the experimental sets containing the bacterial formulation and control sets not treated with bacterial formulation after 45 days of pathogen inoculation. One gram of each soil sample was added in 1 mL of PBS-Tween (phosphate buffered saline, pH 7.0 containing 0.02M phosphate buffer, 0.8% NaCl, 0.02% KCl and 0.05% Tween-20) and mixed by vortexing for 30 seconds. The mixture was allowed to stand at room temperature until the soil particles settled down; the supernatant was filtered using sterile Whatman Grade-I filter paper and the filtrate was used as antigen for indirect ELISA [65].

Indirect ELISA was done as described earlier [34] with necessary modifications. Soil antigen (100 μl) was mixed with an equal volume of 0.2M carbonate buffer (pH 9.6) and coated in the wells of a microtiter plate except the air blank and antigen blank. A homologous antigen-antibody reaction with the whole cell antigen was set as positive control. For this, the bacterial cell suspension in PBS was diluted serially and each dilution was used as antigen for ELISA. For negative control, soil antigen prepared from the control pots containing untreated sterilized and unsterilized soil was allowed to react with ETR17 antiserum. The plate was incubated overnight at 4°C for adsorption. After 24 hour the antigen was poured off, the wells were dried and subsequently washed 4 times with 0.15M PBS (pH 7.2) containing 0.02% sodium azide and 0.05% Tween-20 (v/v). The plate was again air dried and 100 μl of PBS-BSA (0.15M phosphate buffered saline and 1% bovine serum albumin) solution was added to block the unbound sites and incubated for 2 hours at room temperature. The plate was rewashed thoroughly with PBS-Tween and air dried. Two hundred microlitre of antiserum (1:100 dilution; diluted with PBS-Tween) was added to the wells except air blank, antiserum blank and normal serum control where same volume of normal serum was added (1:100 dilution; diluted with PBS-Tween containing 0.5% BSA). Following overnight incubation at 4°C, the sera were poured off and the plate was again thoroughly washed with PBS-Tween and dried. Then, 100 μl of goat anti-rabbit IgG HRP (Horse Raddish Peroxidase) conjugate (1:10,000 dilution) was added to each well except the air blank and antiserum blank and incubated for 2 hours at room temperature. The plate was washed thoroughly with PBS-Tween, air dried and 100 μl of TMB/H_2_O_2_ (1:20), a chromogenic substrate, was added to each well except the air blank. It was incubated for 30 minutes in dark at room temperature for development of blue color due to enzyme-substrate reaction. The reaction was stopped by adding 100 μl of 1N H_2_SO_4_ and absorbance was recorded at 492 nm in an ELISA reader (Mios Junior; Merck, Darmstadt, Germany). A standard curve was plotted using varying bacterial concentrations of the positive control against the corresponding ELISA values and this curve was used to determine the concentration of bacteria in soil. Concentration of bacteria in the serially diluted antigen preparations was determined by NA plate count method.

#### Plant growth promotion by bacterial formulation

To determine the plant growth promoting ability of ETR17, two month old tea seedlings were treated twice at an interval of 15 days by talc formulation of the strain by soil drenching method in sterilized and unsterilized soil sets as done during *in vivo* biocontrol assay. The shoot and root length of the seedlings were measured with a centimeter scale prior to treatment with bacterial formulation and again after 45 days of treatment, by carefully uprooting the seedlings. Plants treated with sterile distilled water grown in both sterilized and unsterilized soil served as control sets. All the treatments were replicated thrice and for each treatment five healthy plants were selected.

### Test for hemolytic activity of ETR17

Hemolytic activity of the bacterial isolate ETR17 was assessed on tryptone soya agar (TSA) medium (Himedia Laboratories, India) supplemented with 5% goat blood. For this, the bacterial isolate was streaked on blood supplemented TSA plates and incubated at 30°C for 48 hours. After incubation, the plate was observed against light for halo formation around the bacterial growth. A clear halo indicates β-hemolysin production, dark colored halo indicate α, α′ hemolysin production and green colored halo indicate γ hemolysin production [66].

### Statistical analysis

Data were analysed by ANOVA using the statistical software SPSS, version 11.0, SPSS Inc., Chicago, Illinois. Specific differences in means were compared by analysing Duncan’s multiple range test (DMRT).

## Results

### *In vitro* screening for antagonistic activity of bacterial isolates

Out of the tested 200 bacterial rhizosphere isolates, 35 strains exhibited antagonism against *L*. *theobromae* in PDA plate dual culture assay. The antagonistic strains were further tested against eight other fungal pathogens of tea. Results revealed that all strains were antagonistic to many of the tested fungi (Table 1). The isolate ETR17 was found to exhibit highest antifungal activity against several of the tested pathogens and was therefore selected for further studies. It was found to limit the growth of fungal mycelia to a considerable extent in comparison to the control. The level of antagonism exhibited by ETR17 in PDA plate assay against individual pathogens is summarized in Table 2.

**Table 1:** List of bacterial isolates from different rhizosphere soil.

Source of isolation	Code assigned	Total No. of isolates	Antagonistic isolates
Bagdogra T.E.	TBD1–10	10	TBD1-7
Bagrakote T.E.	BTRL1-11	11	BTRL6, BTRL8,
Baradighi T.E.	BTR1-24	24	BTR4, BTR8, BTR18,
Diana T.E.	D1-10	10	D6, D7
Ellenbarie T.E.	ETR1-24	24	ETR1, ETR17, ETR20,
Gayaganga T.E.	TGY1–17	17	TGY1, TGY2, TGY4
Good Hope T.E.	GH1-33	30	GH4, GH6, GH12, GH13, GH21.
Kharibarie T.E.	KTR1-18	15	KTR6, KTR18
Labak T.E.	TLB1-11	11	TLB3
Matigara T.E.	TMG1–15	15	TMG1, TMG2, TMG3
Raya T.E.	TR1–20	15	TR1, TR5, TR11, TR19, TR20
Red bank T.E.	TRB1–19	18	TRB1, TRB2, TRB4, TRB7,
**Total number of isolates**		**200**	**35**

T.E.: Tea Estate

**Table 2:** *In vitro* study of antagonistic activity of bacterial isolate ETR17 against fungal pathogens of tea by dual culture test.

Fungal Pathogens	Disease produced	Strain identity	Percent inhibition of pathogen by ETR17 over control*
*Lasiodiplodia theobromae*	Root rot or diplodia	5446.02	51.5 ± 0.8^a^
*Rhizoctonia solani*	Root rot	5995.05	66.7 ± 0.7^b^
*Sphaerostilbe repens*	Violet root rot	SR-01	71.1± 0.7^c^
*Fomes lamaoensis*	Soft rot	FL-01	76.7 ± 0.4^d^
*Ustulina zonata*	Stump rot	UZ-01	61.1 ± 0.5^e^
*Poria hypobrunnae*	Poria root rot and stem canker	PH-01	71.1 ± 0.7^c^
*Pestalotiopsis theae*	Grey blight	PT01	76.7 ± 0.6^d^
*Colletotrichum camelliae*	Brown blight	CC01	65.5 ± 0.5^eb^
*Curvularia eragrostidis*	Leaf spot	4150.2k	81.5 ± 0.5^f^
LSD (at 5%)			2.3

* Inhibition zone was measured when fungal mycelia in the control plate reached the edge of 9 cm petriplates.

Means ± standard error followed by same superscript letters do not differ significantly at P<0.05

### Identification and characterization of antagonistic isolate ETR17

Characterization tests revealed that ETR17 was gram-negative, rod-shaped, facultatively anaerobic bacteria which produced smooth, regular edged, round shaped colonies and excessive red pigment in nutrient agar. It showed catalase positive, oxidase negative and VP positive traits, was fermentative in nature and capable of reducing nitrate, hydrolysing gelatin, was positive for lysine and ornithine decarboxylation and utilizing citrate and different carbohydrates such as glucose, mannitol, sucrose, fructose, inositol, sorbitol, trehalose and xylose. The bacterium was capable of growing over a wide temperature range (4°C to 40°C) with optimum growth temperature of 28–30°C. Comparing the results with the keys published in Bergey’s Manual of Systematic Bacteriology (Palleroni, 1984), it was concluded that the bacteria belonged to the genus *Serratia*. Partial sequence of the 16S rRNA gene (GenBank accession no. JX566992) was 99% similar to such gene sequences of other *S*. *marcescens* strains obtained from GenBank. Results of phylogenetic analyses were consistent with the biochemical and physiological traits and the isolate was therefore designated as *S*. *marcescens*.

### Scanning electron microscopy

**Fig 1:**
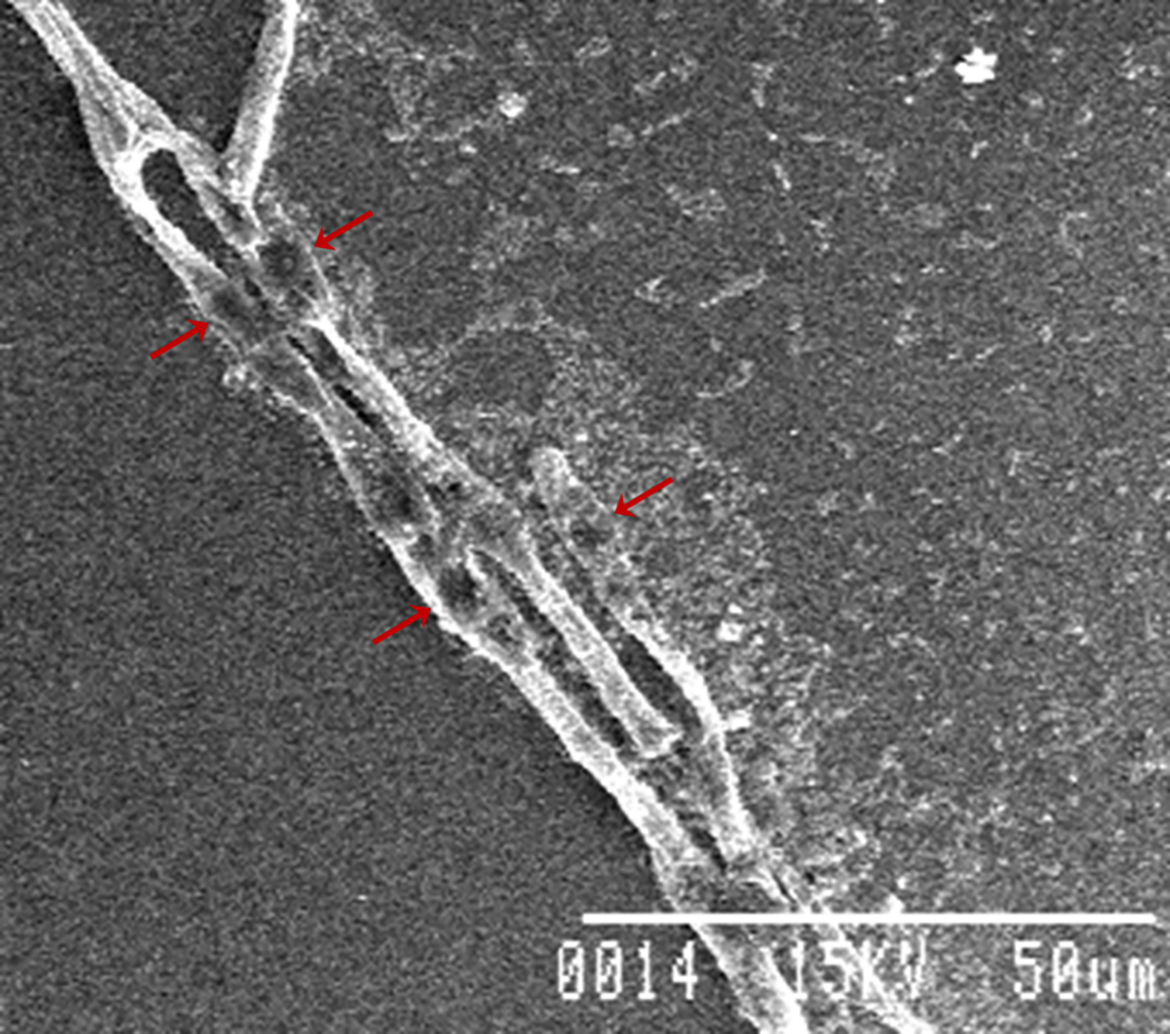
Scanning electron microphotographs showing the antagonistic effect of ETR17 on *Rhizoctonia solani*; lysis of fungal hyphae is indicated by arrows.

For understanding the antagonism involved in the interaction between the fungal pathogen *Rhizoctonia solani* and bacterial strain ETR17, scanning electron microscopy study was conducted. Microscopic observations revealed severe deformations of the fungal mycelia like surface irregularities, perforations, and bulging and bursting in the hyphae at specific sites (Fig 1). Bursting of the fungal mycelia at some places resulted in release of the protoplasm from fungal cell.

### Production of antifungal and plant growth promoting metabolites, hydrolytic enzymes and biofilm

The isolated *S*. *marcescens* strain ETR17 was positive for chitinase, lipase and protease activity while it scored negative for the production of pectinase enzyme and HCN. Additionally, the isolate was capable of producing IAA (40 μg mL^-1^) and siderophore on CAS agar plates but lacked phosphatase activity. Arnow’s test and tetrazolium salt test confirmed the production of both catecholate and hydroxamate type of siderophore. Biofilm formation, which was tested by micro-titre plate assay, depended on the growth medium. It was more pronounced in LB medium (O.D. = 0.254 and 0.448 after 24 and 48 h respectively) than in M9YE medium (O.D. = 0.118 and 0.193 after 24 and 48 h respectively).

### Characterization of antifungal metabolite

**Fig 2:**
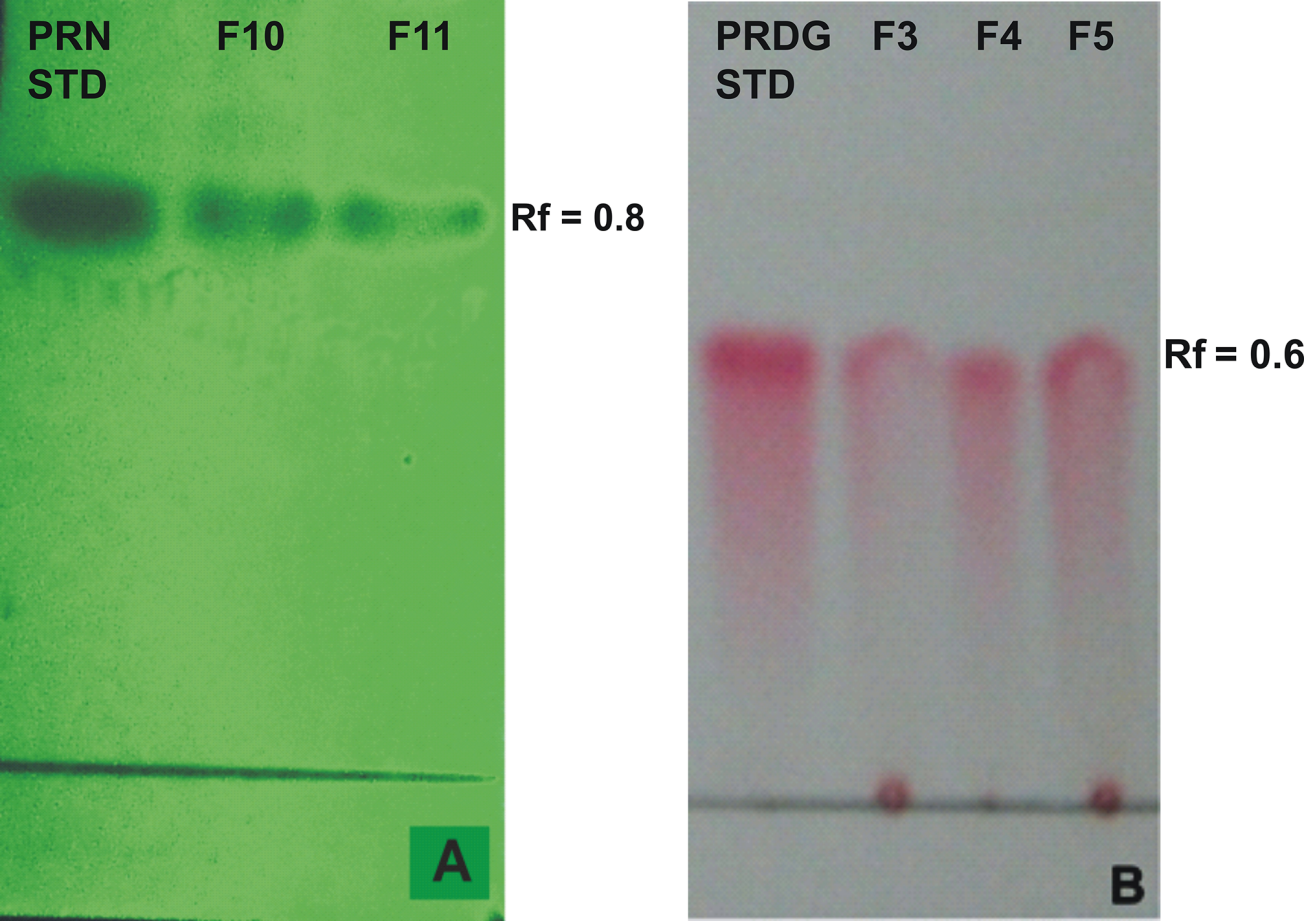
Detection of antibiotics in extracellular culture extract of the antagonistic *S*. *marcescens* strain ETR17 by thin layer chromatography. (A) Pyrrolnitrin detection under UV light (254 nm) in the partially purified silica gel column eluted fractions F10 and F11. (B) Detection of prodigiosin in the partially purified silica gel column eluted fractions F3, F4 and F5 under visible light. [Standard prodigiosin (PDG) and pyrrolnitrin (PRN) are spotted in the extreme left in both (A) and (B)].

**Fig 3:**
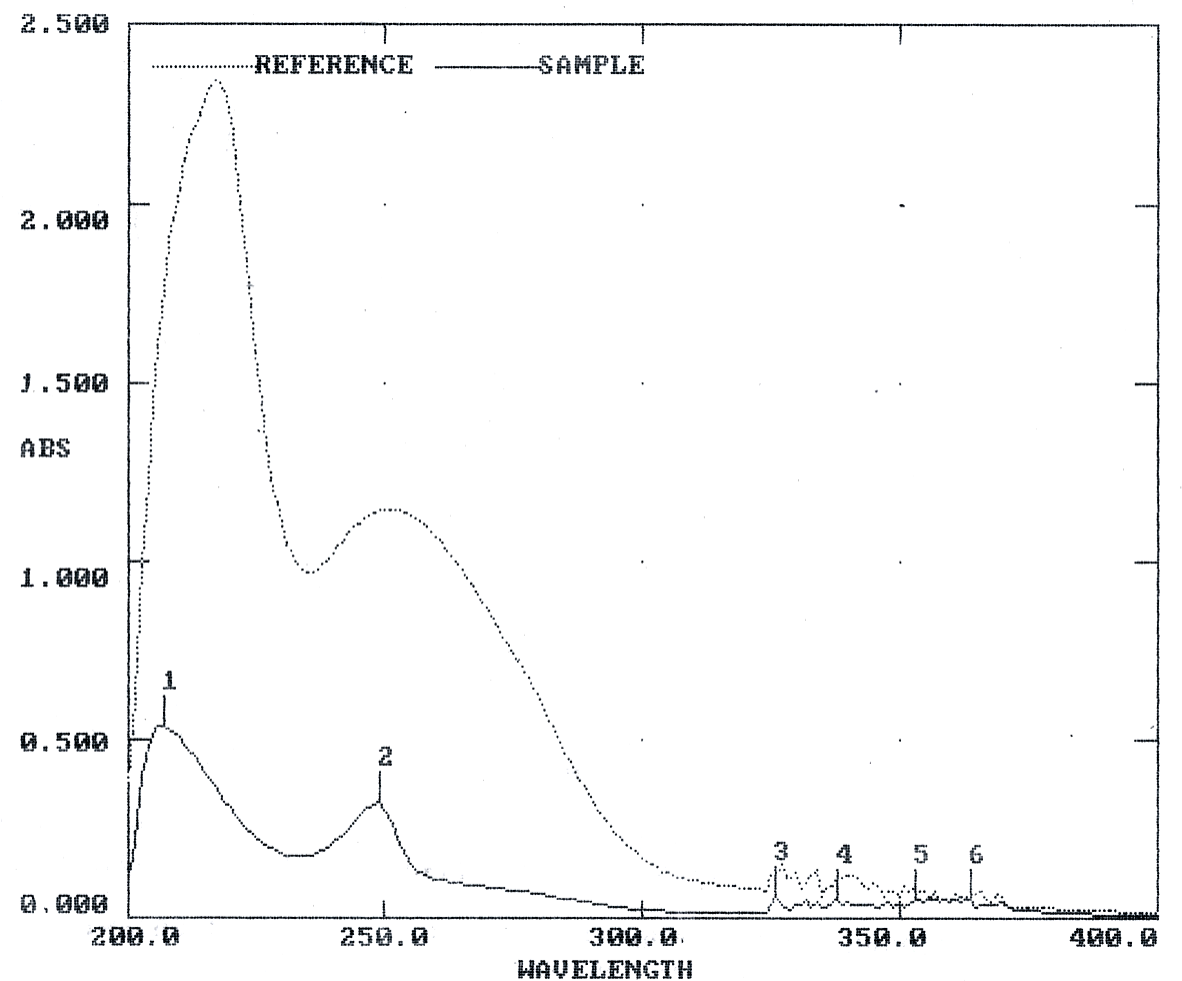
Detection of pyrrolnitrin in extracellular culture extract of the antagonistic *S*. *marcescens* strain ETR17 by UV-spectrophotometric analyses (between 200 nm to 400 nm). Pyrrolnitrin was purified by preparative thin layer chromatography from fraction F10 obtained previously from silica gel column chromatography of the crude culture extract. Two sharp peaks at 210 nm and 249 nm obtained with the partially purified compound were similar to the pyrrolnitrin standard (217 nm and 251 nm).

**Fig 4:**
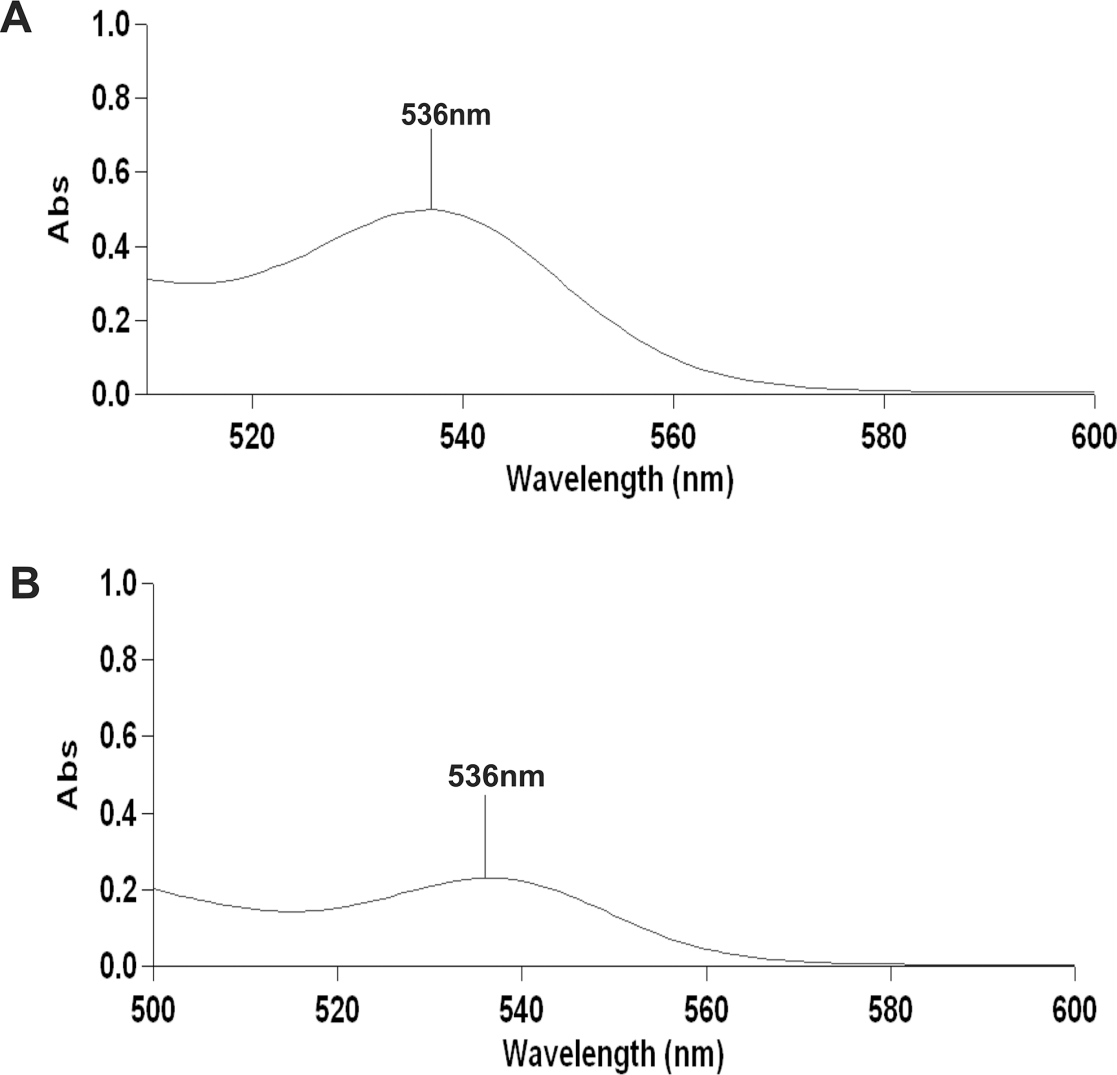
Detection of prodigiosin in extracellular culture extract of the antagonistic *S*. *marcescens* strain ETR17 by VIS-spectrophotometric analyses (between 500 nm to 600 nm). Prodigiosin was purified by preparative thin layer chromatography from fraction F3 obtained previously from silica gel column chromatography of the crude culture extract. (A) A peak at 536 nm obtained with the partially purified compound. (B) Prodigiosin standard having absorbance maxima at 536 nm.

**Fig 5:**
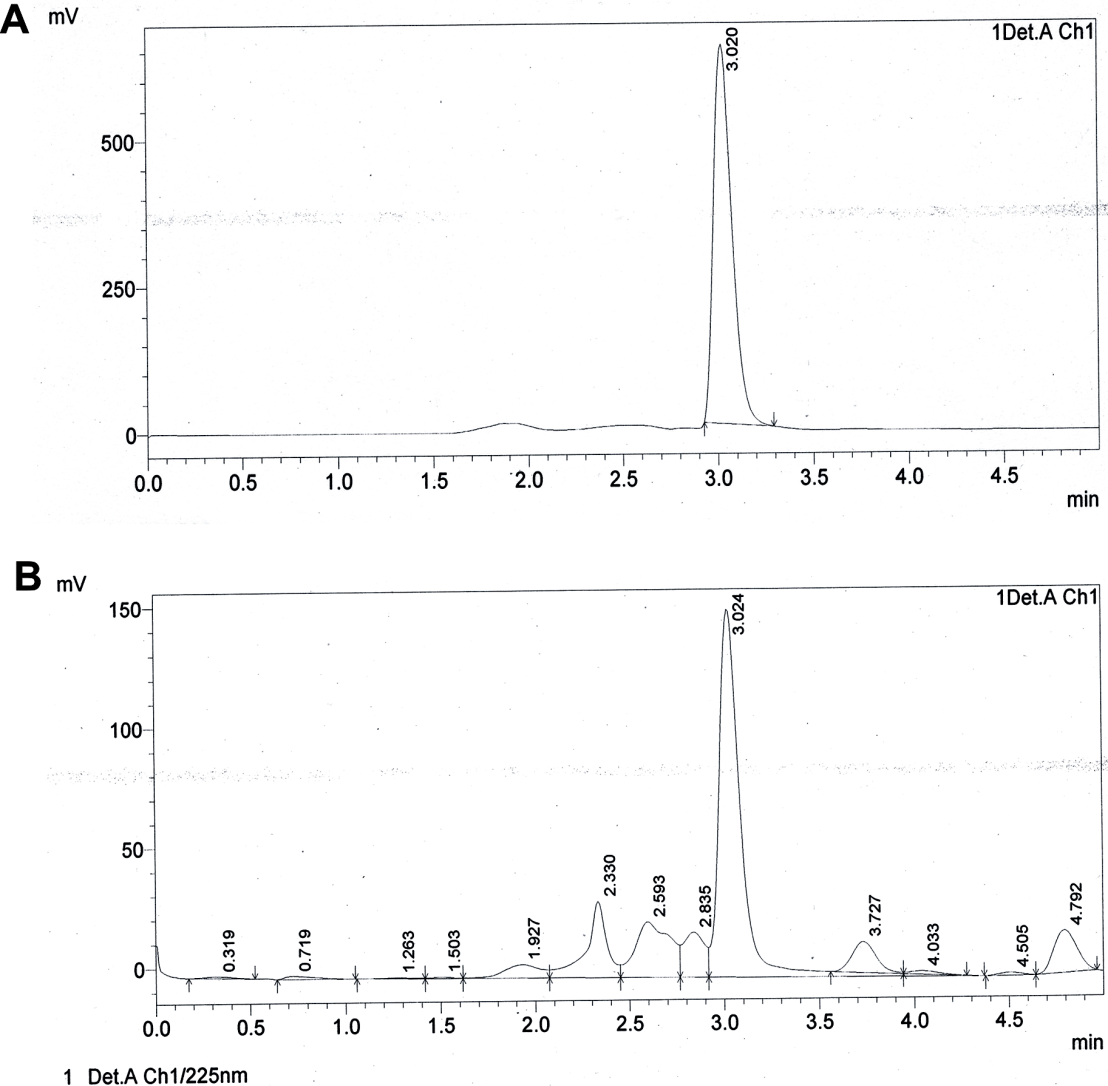
Detection of pyrrolnitrin in extracellular culture extract of the antagonistic *S*. *marcescens* strain ETR17 by high performance liquid chromatography. Chromatograms generated by performing HPLC of the antifungal fraction at 225 nm eluted with 100% methanol (A) chromatogram of pyrrolnitrin standard (retention time = 3.020 min) (B) chromatogram of F10 (45% Ethyl Acetate-55% Petroleum Ether) (retention time = 3.024 min).

**Fig 6:**
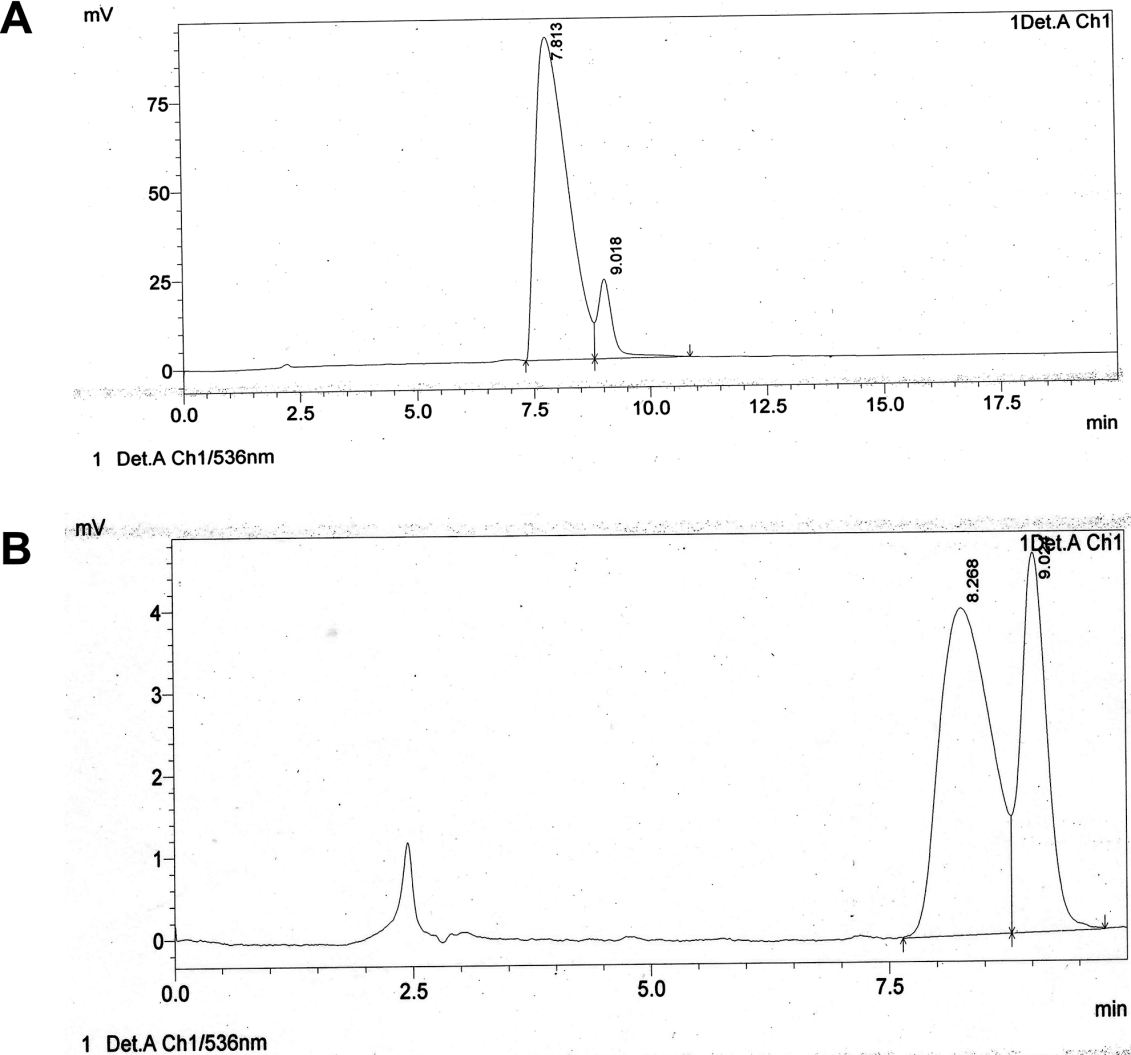
Detection of prodigiosin in extracellular culture extract of the antagonistic *S*. *marcescens* strain ETR17 by high performance liquid chromatography. Chromatograms generated by performing HPLC of the combined bioactive fractions with absorbance maxima of 536 nm (A) chromatogram of prodigiosin standard (1^st^ peak, retention time = 7.813 min; 2^nd^ peak, retention time = 9.018) (B) chromatogram of prodigiosin sample pooled from F3 (10% Ethyl Acetate-90% Petroleum Ether) (1^st^ peak, retention time = 8.266 min; 2^nd^ peak, retention time = 9.02 min).

**Fig 7:**
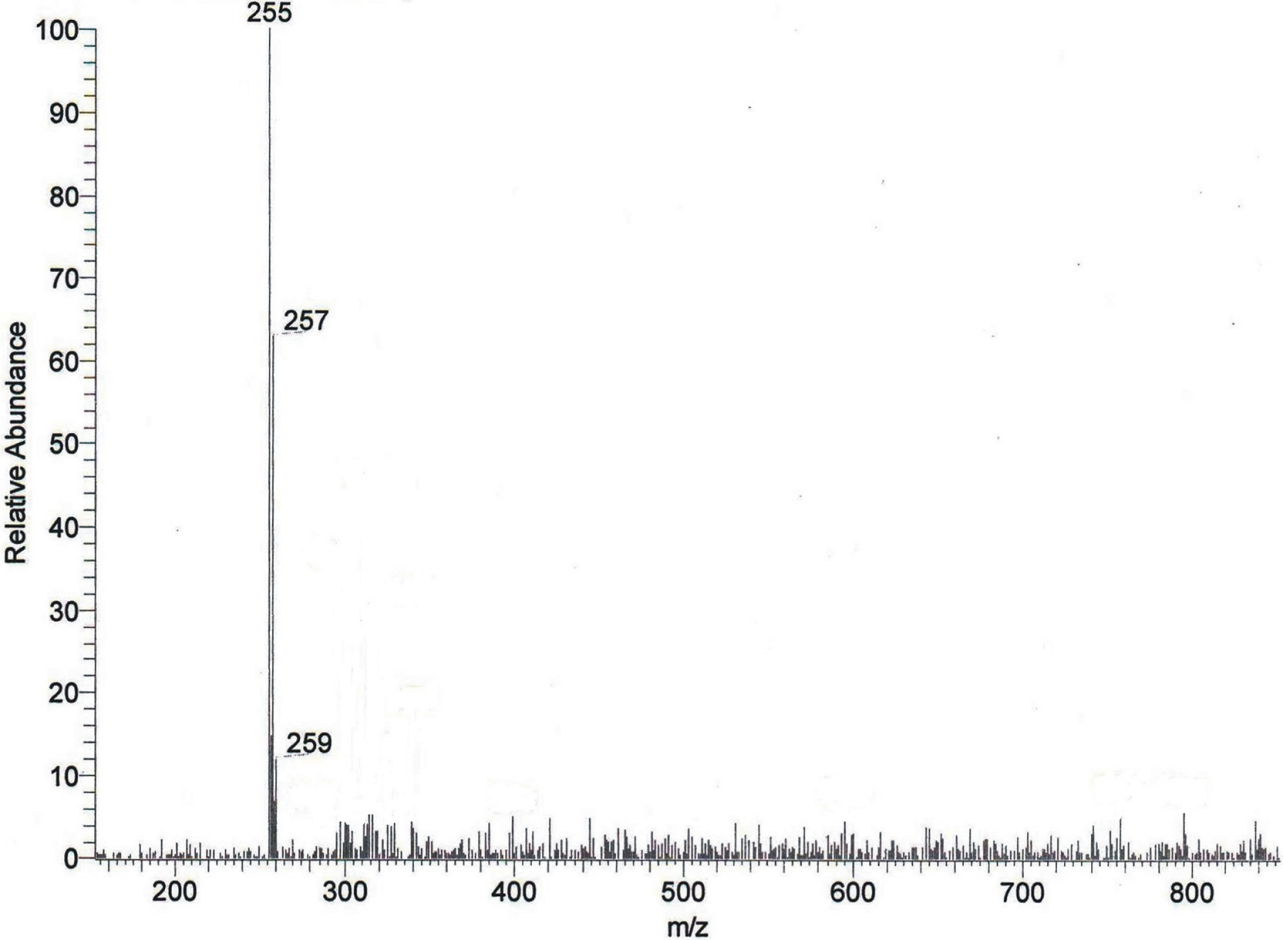
Detection of pyrrolnitrin in extracellular culture extract of the antagonistic *S*. *marcescens* strain ETR17 by liquid chromatography-electrospray ionization-mass spectrometry (LC-ESI-MS) analysis. The combined column fractions F10 and F11 obtained previously from silica gel column chromatography of the crude culture extract was analysed using a gradient elution program with methanol and ammonium acetate buffer at a flow rate of 0.8 mL min^-1^ at 254 nm to obtain a major peak at retention time 14.85 minutes corresponding to pyrrolnitrin. Mass spectra showing molecular ion peak at m/z 259 (M + 2H)^+^ indicating pyrrolnitrin.

The crude extract obtained from the culture media produced clear zones of inhibition around the wells against the test pathogens indicating antibiotic production by ETR17. Column chromatography of the crude extract and subsequent bioassay of the fractions revealed that the fractions F3, F4, F5, F10 and F11 possessed antifungal activity. Comparative analysis of each bioactive fraction with standard antibiotics in TLC plates under UV light (254 nm) revealed that the major compound in fractions 10 and 11 produced deep green colored spots at R_f_ = 0.80 which was similar to the spot produced by standard pyrrolnitrin (Fig 2A). Prodigiosin was detected in fractions F3, F4 and F5 at R_f_ = 0.6 which produced a visible reddish pink band which was same as the standard (Fig 2B). Phenazine was not detected in any of the fractions. UV-VIS scan revealed that the fractions F10 and F11 produced two sharp peaks at 210 nm and 249 nm (Fig 3) which was similar to the standard pyrrolnitrin (217 nm and 251 nm). Fractions F3, F4 and F5 produced a sharp peak at 536 nm (visible region) which corresponded to the prodigiosin standard (Fig 4). Comparison of the HPLC profiles obtained for the purified compounds recovered from preparative TLC plates with the profiles of standard antibiotics revealed that a single major peak, which was obtained with the fractions F10 and F11 at retention time (RT) of 3.024 min and 3.022 min respectively was comparable to standard pyrrolnitrin (RT: 3.020 min) (Fig 5). The antibiotic, purified from fractions F3, F4 and F5 by preparative TLC, were combined prior to HPLC analysis. The sample produced two major peaks (RT: 8.268 min and 9.024 min) which was similar to that of the standard prodigiosin (RT: 7.813 min; and 9.018 min) (Fig 6). LC-ESI-MS analysis of the combined bioactive fractions F10 and F11 obtained by column chromatography revealed molecular ion peak at m/z 259 (M + 2H) ^+^ (Fig 7). This confirmed the presence of pyrrolnitrin in the extracellular culture extract of *S*. *marcescens* ETR17.

### Biocontrol of root rot disease in tea

**Fig 8:**
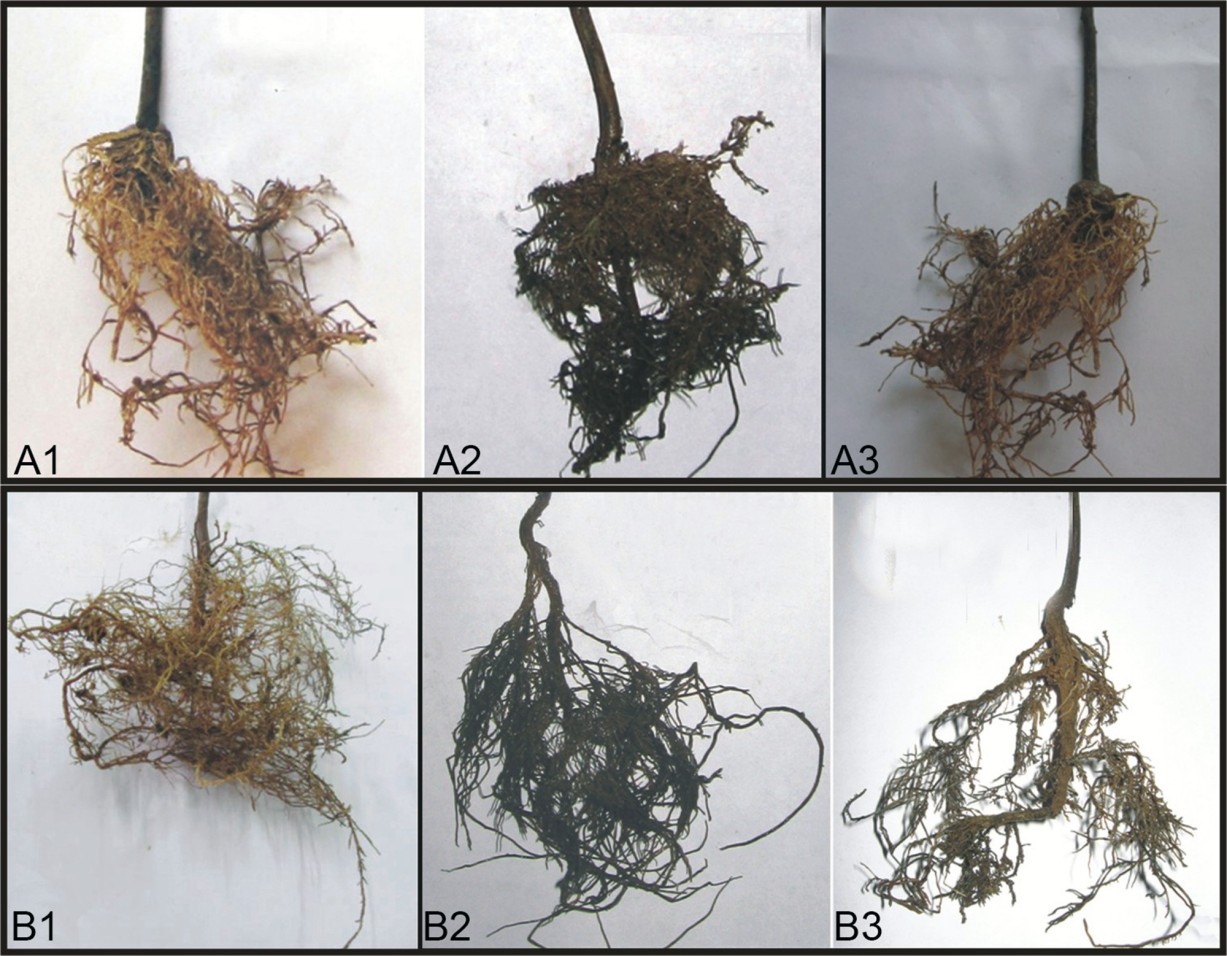
Root rot disease occurrence in tea (TS-520 variety) after 30 days of treatment by talc based formulation prepared with ETR17 in (A1) sterile soil ETR17 treated (A2) sterile soil untreated control (A3) sterile soil fungicide treated and (B1) unsterile soil ETR17 treated (B2) unsterile soil untreated control (B3) unsterile soil fungicide treated.

Treatment of tea seedlings with talc based formulation prepared from the isolate ETR17 three days prior to inoculation with the root rot pathogen *R*. *solani* at 10 g per kg soil reduced the disease development significantly under both sterile and non-sterile soil conditions when compared to control (Table 3). In contrast, the progression of disease remained unhindered and finally at 45 dapi, severe disease symptoms appeared in the control plants that were not treated with either ETR17 or thiophanate methyl but inoculated with the pathogen. The control set therefore recorded a high mean disease index value of 78.3 and 81.7 under sterile and non-sterile soil conditions respectively. However, in the ETR17 treated sets, the mean disease index increased slowly and reached its highest on 30^th^ day (16.7 and 20.0 under sterile and non sterile conditions respectively) after which the roots showed signs of recovery which caused the disease index to decrease marginally (15.0 and 18.3 under sterile and non-sterile conditions respectively) by the 45^th^ day. The fungicide recorded lower efficacy of disease control than ETR17 and also did not show any recovery during the period of observation. No significant difference in the overall efficacy of disease control between sterile (80.8%) and non-sterile (77.6%) soil conditions was noted over 45 dapi (Fig 8).

**Table 3:** Effect of powder formulation of biocontrol bacterial isolate ETR17 on root-rot disease incidence of tea plants (TS-520) during *in vivo* study.

Treatments	Mean disease index					PEDC*
	15 dapi**	20 dapi	25 dapi	30 dapi	45 dapi	45 dapi
Control (Sterilized soil)	26.7±0.9^a^	36.7±1.0^a^	58.3±0.9^a^	70.0±0.9^a^	78.3±0.9^a^	0
Control (Unsterilized soil)	28.3±0.4^a^	50.0±0.8^b^	63.3±0.7^b^	73.3±0.5^b^	81.7±0.9^a^	0
Fungicide (Sterilized soil)	0	3.3±0.3^c^	12.5±0.3^c^	23.3±0.7^ce^	25.0±0.6^b^	68.1±0.5^b^
Fungicide (Unsterilized soil)	0	6.7±0.4^dc^	20.0±0.7^d^	25.0±0.7^c^	28.3±0.8^b^	65.4±0.5^b^
ETR17 (Sterilized soil)	0	5.0±0.5^dc^	13.3±0.6^ec^	16.7±0.7^d^	15.0±0.9^c^	80.8±0.8^c^
ETR17 (Unsterilized soil)	0	8.3±0.6^d^	15.0±0.8^d^	20.0±0.4^ed^	18.3±0.5^c^	77.6±0.6^d^
LSD	1.3	2.0	2.2	2.1	2.4	1.6

*PEDC = Percent efficacy of disease control

**dapi = days after pathogen inoculation

Means ± standard errorfollowed by same superscript letters do not differ significantly at P<0.05

### Plant growth promotion by bacterial formulation

Tea seedlings treated with talc based formulation of the biocontrol bacteria showed approximately 50% higher growth response under both sterile and non-sterile sterile conditions when compared to the untreated controls (Table 4). An increase in shoot and root length of tea seedlings by 6.0 cm and 4.7 cm respectively was recorded in treated plants under sterile soil conditions after 45 days of plantation. But in untreated control plants, shoot length increased by 3.0 cm and root length increased by 2.5 cm only under similar conditions. Similarly, under non-sterile conditions, significantly better shoot and root growth was recorded by the ETR17 treated plants than the respective control.

**Table 4:** Effect of powder formulation of biocontrol bacterial isolate ETR17 on the growth of tea plants (TS-520) during *in vivo* study.

Treatment	Plant response					
	Shoot length (cm)		Increase in shoot length (cm)	Root length (cm)		Increase in root length (cm)
	(0 day)	(45 days)		(0 day)	(45 days)	
Control (Sterilized soil)	12.0±0.5^a^	15.0±0.4^a^	3.0 ±0.3^a^	7.5 ±0.3^a^	10.0±0.3^a^	2.5 ±0.3^a^
Control (Unsterilized soil)	11.6±0.3^a^	15.0±0.6^a^	3.4 ±0.5^a^	10.0 ±0.5^a^	12.0 ±0.4^ab^	2.0±0.5^a^
ETR17 (Sterilized soil)	12.0±0.4^a^	18.0±0.5^b^	6.0±0.4^b^	8.5±0.2^a^	13.2 ±0.4^b^	4.7 ±0.4^b^
ETR17 (Unsterilized soil)	12.8±0.5^a^	19.2±0.3^b^	6.4±0.3^b^	10.0±0.7^a^	13.8±0.5^b^	3.8±0.2^ab^
LSD (0.5%)	1.4	1.5	1.2	1.5	1.3	1.2

Means ± standard error followed by same superscript letters do not differ significantly at P<0.05

### Survivability of bacterial cells in talc formulation

**Fig 9:**
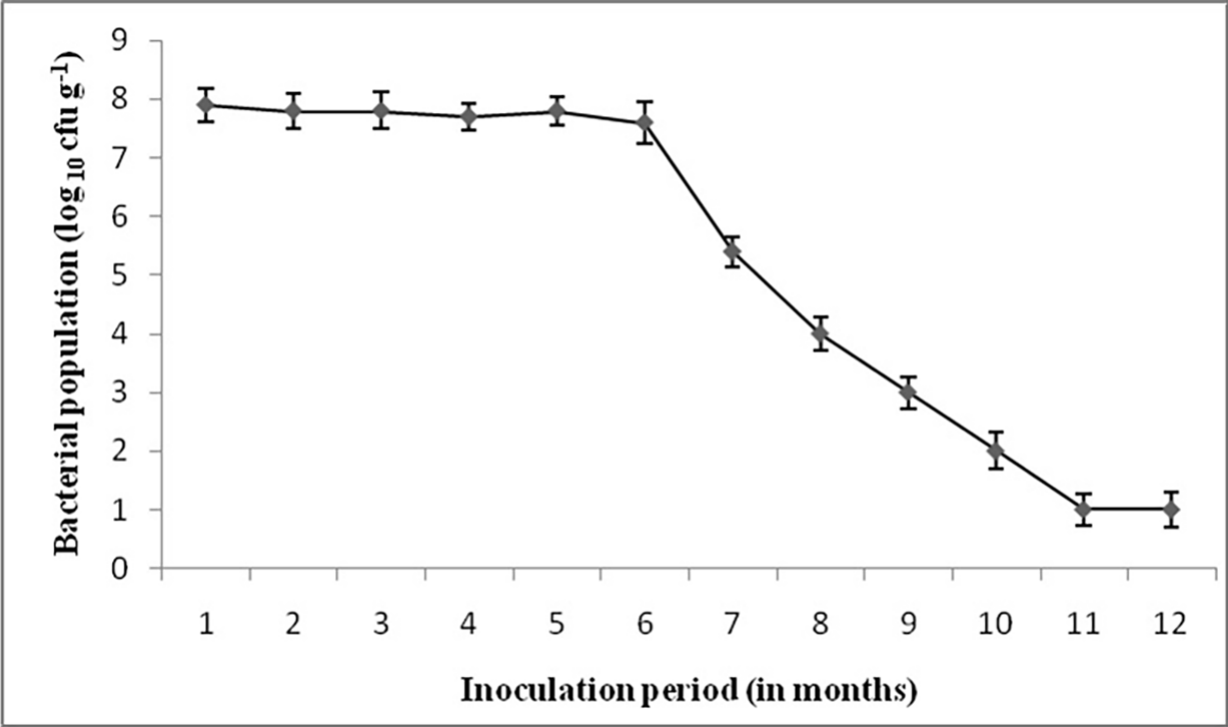
Viability of *S*. *marcescens* ETR17 isolate in talc formulation. The bacterial cell population was measured for 12 months at 1 month time interval.

Survivability of ETR 17 cells in the talc based formulation was tested every one month for a period up to one year. The bacterial cell population remained almost stationary for the first six months with no significant changes when stored at room temperature. A reduction in bacterial count was noticed at the 7^th^ month and this reducing trend continued until one year (Fig 9).

### Sustainability of ETR17 in the rhizosphere

Results (Table 5) showed that the soil population was high after 45 days of application of the bacteria as formulation. The bacterial concentration was estimated to be 8x10^8^ cfu g^-1^ and 9x10^8^ cfu g^-1^ in the sterilized and unsterilized soil respectively. The polyclonal antibody raised against ETR17 showed negligible cross reactions as evident from the low ELISA values obtained with the unsterile soil antigen (untreated negative control).

This study confirmed the presence of ETR17 in tea rhizosphere on application of talc formulation in both sterilized and unsterilized soil sets for at least 45 days.

**Table 5:** Determination of *S*. *marcescens* ETR17 concentration in soil by ELISA using soil antigens prepared from tea rhizosphere soil, treated with biocontrol formulations and ETR17 antiserum after 45 days of pathogen inoculation^a^.

Treatment	Absorbance at 492nm	Bacterial concentration (cfu/g soil)^c^
Control (Sterilized soil)	0.010	0
Control (Unsterilized soil)	0.166	1.0 x 10^8^
ETR17 (Sterilized soil)	0.581	8.0 x 10^8^
ETR17 (Unsterilized soil)	0.660	9.0 x 10^8^
Positive control^b^ ETR17 (whole cell)	0.744	Bacterial concentration (cfu/mL) 1.2 x 10^9^

^a^ETR17 application was done three days prior to pathogen inoculation. Primary antiserum dilution: 1:100; secondary antibody (goat anti-rabbit IgG-HRP conjugate) dilution: 1:10,000.

^b^Whole cell antigen prepared with bacterial cells cultured in NB for 48 hours served as positive control.

^c^Bacterial concentrations were determined using standard curve plotted using varying bacterial concentrations against the corresponding ELISA values of the positive control

### Hemolytic activity

The test for hemolytic activity on blood supplemented TSA plates revealed that the isolate ETR17 lacked the hemolytic activity since no halo formation occurred around the bacterial colonies.

## Discussion

A total of 200 bacterial strains were isolated from tea rhizosphere from 15 tea growing fields, among which 35 strains showed antifungal activity. The isolate ETR17, which showed best antagonistic activity against eight fungal pathogens of tea, was identified as *S*. *marcescens* after analyzing both the phenotypic characters and 16S rRNA gene sequence. *S*. *marcescens* strains unlike the *S*. *plymuthica* and *S*. *liquefaciens* does not produce gas in glucose, shows lysine and ornithine decarboxylation but not arginine decarboxylation and does not ferment L-arabinose and xylose [67]. Additionally, the 16S rRNA gene sequence of ETR17 showed 99% similarity with other *S*. *marcescens* gene sequences available in the NCBI GenBank which confirmed the phenotypic data.

Production of antifungal metabolites by biocontrol agents is strongly correlated with their biocontrol activity. Chitinases, which had earlier been considered as a major factor of biocontrol [68] along with other hydrolytic enzymes, degrade fungal cell walls and inhibit fungal growth [69, 70]. ETR17 produced chitinase, protease and lipase but did not produce cellulose and pectinase. *Serratia* is one of the most competent producers of chitinases, whose production, activity and gene regulation are well studied [71]. Purified chitinases from *S*. *plymuthica* [72], *S*. *proteamaculans* [73] and *S*. *marcescens* [74] are reported to be antagonistic to different pathogenic fungi. But in spite of the strong antifungal activity, factors other than chitinases are considered more essential for biocontrol of plant diseases [75] indicating that chitinases may form only a part of the multi-component mechanism which underlies biocontrol by *Serratia* strains in the environment [76]. Inability of ETR17 to produce pectinase and HCN may be regarded as an advantageous trait because the production of pectinase is considered as an undesirable feature of plant beneficial bacteria and HCN inhibits plant growth and yield due to the interference with cytochrome oxidation [48, 56].

The role of iron-chelating siderophore produced by the rhizobacteria in biocontrol is well documented [7, 77]. Siderophore producing antagonistic bacteria like *Serratia*, *Bacillus*, *Pseudomonas* etc. exhibit better rhizosphere competence and suppress plant disease by inhibiting the growth or the metabolic activity of plant fungal pathogens by sequestering iron since fungal siderophores have lower affinity [78, 79, 80]. The present isolate was capable of producing both catecholate and hydroxamate type of siderophore. Additionally, ETR17 produced IAA, which is considered as the best-known hormone produced by the plant-associated microorganisms [81].

The nature of antagonism involved in the interaction between ETR 17 and *R*. *solani* was observed by scanning electron microscopy. The fungal mycelium at the interaction zone in dual cultures was distorted, showing bulging, lysis and bursting of the hyphae. Abnormal hyphal structures caused due to cell wall degradation were prominent. Ordentlich et al. [82] also observed severe mycelial deformities like holes in the hyphae at the interaction zone of bacterial antagonist *S*. *marcescens* and the pathogen *Sclerotium rolfsii*. The mycelial and spore deformities of *Aspergillus parasiticus* NFRI-95 caused by *S*. *marcescens* JPP1 crude culture filtrate was observed under SEM by Wang et al. [74]. Enzymatic degradation of fungal cell walls leading to loss of protoplasm is considered as a major antagonistic mechanism involved in biocontrol activity of many bacteria [83, 84]. The present strain produced several lytic enzymes, particularly chitinase, responsible for lysis of fungal cell wall. However, further studies are required to ascertain the exact mechanism used by this strain for its biocontrol action. Biofilm production, observed in several biocontrol bacteria, is considered to be a beneficial feature for effective colonization on root surface [85–88]. The present strain ETR 17 formed biofilm when tested after 24 and 48 h of incubation. Better biofilm production was noted in LB medium compared to M9YE medium. Biofilm formation was found to vary depending on the medium in *S*. *plymuthica* HRO-C48 [22].

Further studies on biological basis of the antagonism shown by ETR17 revealed that the strain produced the antibiotics pyrrolnitrin and prodigiosin. These were initially detected in the culture filtrate extract by TLC where they showed same migration as the standards. Spectroscopic analysis of the column fractionated extract of the culture filtrate further confirmed the presence of these antibiotics. Pyrrolnitrin is a strong antifungal antibiotic produced by a narrow range of gram negative bacteria such as *Pseudomonas*, *Burkholdaria*, *Serratia* and *Enterobacter* [88–90]. It has been correlated with the potential of some of these bacteria to control fungal diseases in plants [88, 17]. Pyrrolnitrin has been reported from *Serratia marcescens* having absorbance maxima between 210–225 nm and being involved in pathogen suppression [91, 16]. Pyrrolnitrin from particular *Serratia* strains have been shown to be directly linked to its *in vitro* antifungal activity [92], biocontrol capacity [16] and more important for the antifungal action than its other antagonistic properties [70]. Transcriptional response studies with biocontrol *Serratia* strain grown in presence of *R*. *solani in vitro*, revealed significantly increased expression of genes related to the biosynthesis of the antibiotic pyrrolnitrin [93]. The other antibiotic, prodigiosin, detected during this study in the culture filtrate of ETR17, is well known as the tripyrrole red pigmented antibiotic that is produced by three species of *Serratia* namely *S*. *marcescens*, *S*. *rubidaea* and *S*. *plymuthica* [94]. It contributes to survival under competition and possesses antifungal, antibacterial, antiprotozoal, immunosuppressive and anticancer properties [76]. Prodigiosin purified from *S*. *marcescens* controlled Black Sigatoka in banana by a synergistic action with purified chitinases [95].

Results of the glasshouse experiments conducted by application of talc based formulation of ETR17 were consistent with the *in vitro* studies. Incidence of root rot in bacteria treated tea plants were considerably lower in comparison to untreated control as well as the fungicide treated sets. The bacterial population in the bioformulation remained unchanged upto six months of storage. Additionally, ETR17 formulation also increased the root and shoot length of the tea seedlings under both sterile and unsterile soil conditions in comparison to the untreated controls. *Serratia* species are known for their ability to produce a multitude of secondary metabolites and extracellular enzymes that directly contribute to establish themselves by outcompeting other microorganisms when colonizing their respective niche [96]. There are reports on the biocontrol activity of *S*. *marcescens* against *R*. *solani* causing damping off in cyclamen [97]; *Phytophthora parasitica* causing root rot disease in citrus [98]; *Pyricularia oryzae* causing blast disease in rice [28]; *Fusarium oxysporum* causing wilt in Banana [99]; and *Fomes lamaoensis* causing brown root rot in tea [100]. Our strain produced chitinase, siderophore and multiple antibiotics *in vitro* but further studies are required to confirm how these factors either individually or synergistically were actually involved in the biocontrol action *in vivo*. Characterization of biocontrol strains most often includes evaluation of plant growth promoting traits whose presence increases the overall quality of a strain [76]. *S*. *marcescens* have been used widely in several studies in a wide range of crops but such reports involving tea are few. In a previous study, a phosphate solubilizing strain of *S*. *marcescens* promoted growth and induced resistance in tea plants against brown root rot caused by *Fomes lamaoensis*. Our strain did not show phosphate solubilizing ability; but it still promoted plant growth which was not unexpected as the bacteria showed IAA production during *in vitro* tests. However, further studies are necessary to confirm whether IAA was responsible for the growth promotion in tea plants. Other studies have shown that *Serratia* strains possessed growth promoting attributes such as phosphate solubilizing ability and IAA production and exhibited beneficial effect on several plants such as coconut, paddy, cowpea and chickpea [101, 102].

Sustainability of ETR17 in the soil environment when applied as talc based formulation was studied by ELISA. ELISA is considered more reliable compared to other techniques because the raised polyclonal antiserum shows no non-specific reactions with other bacterial species in the environment [103]. Literature studies suggest that although ELISA was used for studying the presence of several bacterial populations in soil and bacteria-plant root associations but the use of this technique for detection of biocontrol strain *S*. *marcescens* in soil upon *in vivo* application was done for the first time in our study. Results showed that ETR 17 population remained high in the soil in both sterilized and unsterilized soil treatments for 45 days. In a previous study, *S*. *marcescens* B2 which suppressed rice sheath blight caused by *Rhizoctonia solani* remained in rhizosphere soil at a concentration of 10^8^ cfu g^-1^soil for more than 4 weeks under glass house conditions [104]. The present strain ETR17 remained in soil at a concentration of 8x10^8^ cfu g^-1^ and 9x10^8^ cfu g^-1^ in the sterilized and unsterilized soil respectively which shows that this strain has potential as an effective and persistent biological control agent for root rot in tea.

Some of the bacterial biocontrol agents including *Serratia* can enter into mutualistic interactions with plant and human hosts [105, 106]. Therefore, it is essential to appraise the risk of every potential biocontrol bacteria in order to avoid using potential human pathogens as agricultural inputs. Pathogenic strains of *Serratia* are reported to produce hemolysins [107, 108], therefore hemolytic activity was tested in this study which showed negative result.

## Conclusions

A pyrrolnitrin and prodigiosin producing *S*. *marcescens* strain ETR17, which was isolated from tea rhizosphere was effective in suppressing root rot disease caused by *R*. *solani* in tea plants. The isolate exhibited biocontrol traits such as broad spectrum antifungal activity and production of lytic enzymes, HCN, IAA, siderophore and antibiotics. The talc formulations of the bacterial isolate significantly reduced the root rot disease in tea and stimulated plant growth *in vivo*. In addition, the isolate did not show hemolytic activity which is a general feature of pathogenic *S*. *marcescens* strains. Therefore, after considering the safety and biocontrol potential of ETR17, it may be suggested that the bacterium can be used in management of fungal infections of tea after field trials. The use of this bacterial strain as a biocontrol agent to increase plant protection seems to be a promising approach for controlling fungal diseases, as compared to contemporary chemical agents used in agriculture.

## Supporting information

S1 Table *In vitro* study of antagonistic activity of bacterial isolates against *L*. *theobromae* by dual culture test (initial screening). (DOCX)
