# Supplementary material for: Evaluation of the biocontrol efficacy of a Serratia marcescens strain indigenous to tea rhizosphere for the management of root rot disease in tea
Source: PLoS One. 2018 Feb 21;13(2):e0191761. doi: 10.1371/journal.pone.0191761 (PMC5821441; doi:10.1371/journal.pone.0191761)
Supplement: S1 Table — (DOCX) [file pone.0191761.s001.docx]

| Antagonists | % inhibition of *L. theobromae* over control |
| --- | --- |
| BTR4 | 33.3 ± 1.2 |
| BTR8 | 43.3 ± 1.4 |
| BTR18 | 45.5 ± 1.3 |
| BTR19 | 43.3 ± 1.3 |
| BTR21 | 37.8 ± 1.5 |
| BTR22 | 20.0 ± 1.4 |
| BTR23 | 22.2 ± 1.3 |
| BTRL6 | 44.4 ± 1.1 |
| BTRL8 | 40.0 ± 1.3 |
| BTRL9 | 33.3 ± 1.2 |
| BTRL11 | 38.9 ± 1.6 |
| D6 | 35.5 ± 1.2 |
| D7 | 36.7 ± 1.6 |
| D8 | 32.2 ± 1.0 |
| ETR1 | 48.9 ± 1.5 |
| ETR17 | 51.5 ± 0.8 |
| ETR20 | 34.4 ± 1.5 |
| ETR24 | 44.4 ± 1.6 |
| GH4 | 34.4 ± 1.5 |
| GH6 | 21.1 ± 1.5 |
| GH12 | 31.1 ± 1.2 |
| GH13 | 32.2 ± 1.8 |
| GH21 | 33.3 ± 1.1 |
| GH22 | 46.7 ± 1.3 |
| GH27 | 38.9 ± 1.5 |
| GH32 | 27.8 ± 1.5 |
| KTR6 | 47.8 ± 1.7 |
| KTR18 | 21.1 ± 1.6 |
| TLB3 | 32.2 ± 1.3 |
| TBD7 | 34.4 ± 1.6 |
| TMG1 | 20.0 ± 1.4 |
| TMG2 | 11.1 ± 1.2 |
| TMG3 | 33.3 ± 1.2 |
| TMG7 | 20.0 ± 1.1 |
| TRB1 | 43.3 ± 1.0 |
| TRB2 | 35.5 ± 1.4 |
| TRB4 | 31.1 ± 1.2 |
| TRB7 | 30.0 ± 1.1 |
| TRB12 | 20.0 ± 1.3 |
| TRB14 | 28.9 ± 1.2 |
| TRB18 | 30.0 ± 1.3 |
| TR1 | 34.4 ± 1.4 |
| TR5 | 33.3 ± 1.2 |
| TR11 | 26.7 ± 1.5 |
| TR19 | 22.2 ± 1.3 |
| TR20 | 13.3 ± 1.2 |
| TGY1 | 26.7 ± 1.6 |
| TGY2 | 13.3 ± 1.6 |
| TGY4 | 11.1 ± 1.5 |
| TGY6 | 18.9 ± 1.6 |
| TGY7 | 17.8 ± 1.5 |
